# Immune infiltrates as predictive markers of survival in pancreatic cancer patients

**DOI:** 10.3389/fphys.2013.00210

**Published:** 2013-08-09

**Authors:** Maria Pia Protti, Lucia De Monte

**Affiliations:** ^1^Tumor Immunology Unit, Transplantation and Infectious Diseases, San Raffaele Scientific InstituteMilan, Italy; ^2^Division of Immunology, Transplantation and Infectious Diseases, San Raffaele Scientific InstituteMilan, Italy

**Keywords:** pancreatic cancer, tumor infiltrating lymphocytes, macrophages, mast cells, survival predictive factor, univariate and multivariate analyses

## Abstract

Pancreatic cancer is a devastating disease with dismal prognosis. The tumor microenvironment is composed by multiple cell types, molecular factors, and extracellular matrix forming a strong desmoplastic reaction, which is a hallmark of the disease. A complex cross-talk between tumor cells and the stroma exists with reciprocal influence that dictates tumor progression and ultimately the clinical outcome. In this context, tumor infiltrating immune cells through secretion of chemokine and cytokines exert an important regulatory role. Here we review the correlation between the immune infiltrates, evaluated on tumor samples of pancreatic cancer patients underwent surgical resection, and disease free and/or overall survival after surgery. Specifically, we focus on tumor infiltrating lymphocytes (TILs), mast cells (MCs) and macrophages that all contribute to a Th2-type inflammatory and immunosuppressive microenvironment. In these patients tumor immune infiltrates not only do not contribute to disease eradication but rather the features of Th2-type inflammation and immunosuppression is significantly associated with more rapid disease progression and reduced survival.

## Introduction

A relationship between tumors and immune system exists (Schreiber et al., 2011). Indeed, multistep carcinogenesis results from a cross-talk between cancer-cell-intrinsic factors and host immune system (cell-extrinsic) effects (Zitvogel et al., 2006). This cross-talk leads to different outcomes that are well explained by the concept of the three Es of cancer immunoediting (Dunn et al., 2004). At early stages immunosurveillance is responsible for tumor rejection (Elimination phase), in advanced stages the immune system prevents tumor outgrowth and edits tumor immunogenicity (Equilibrium phase) with the appearance in late stages of tumor cell variants that are no longer recognized by the immune system but rather tumors develop strategies to redirect infiltrating immune cells toward a pro-tumorigenic phenotype (Escape phase) (Dunn et al., 2004; Schreiber et al., 2011). The mechanisms of immune escape have been recently recognized (Hanahan and Weinberg, 2011; Hanahan and Coussens, 2012) as an emerging hallmark of cancer.

Tumors are complex organs composed by tumor cells as well as a variety of cells and factors forming the tumor microenvironment. Cells present in the tumor microenvironment are cancer associated fibroblasts (CAFs), endothelial cells, pericytes and immune cells, among which macrophages, dendritic cells (DCs), natural killer cells, mast cells (MCs), granulocytes, B cells and naïve and memory T cells [including cytotoxic CD8^+^ T cells and different subsets of CD4^+^ T and regulatory T cells (Tregs)]. All these cell types and their released factors interact with each other and determine the cytokine/chemokine milieu, which ultimately have an impact on tumor regression or progression.

Studies in several tumors have evaluated the association between anti-tumor immunity and cancer prognosis but only in recent years the development of more accurate methods for analysis of immune infiltrates has allowed the identification of the features of productive anti-tumor immunity (Fridman et al., 2011). Large-scale studies have then revealed the prognostic and predictive impact of immune infiltrates (Pages et al., 2005; Galon et al., 2006; Denkert et al., 2010) and international efforts have been put together to standardize predictive immune scores for prognosis in several tumor histotypes (Galon et al., 2012).

Pancreatic ductal adenocarcinoma (PDAC) is a very aggressive disease with dismal prognosis (Hidalgo, 2010). The tumor microenvironment is characterized by a strong desmoplastic reaction, which is a hallmark of the disease and it is believed to play a role in carcinogenesis and in tumor progression through its effects on angiogenesis, resistance to therapy and metastatic spread of tumor cells (Kleeff et al., 2007; Erkan et al., 2012). Fibrosis is due to activation by tumor and immune cells of pancreatic stellate cells, which are responsible for extracellular matrix deposition. Importantly, fibrogenesis is differentially regulated by Th1 (i.e., IFN-γ) and Th2 (i.e., IL-4, IL-5, and IL-13) cytokines, which exert opposing roles by promoting collagen degradation and synthesis, respectively (Wynn, 2004). Th1 or Th2 polarized immune cells may thus differentially contribute to fibrosis in PDAC and possibly influence tumor progression.

The role of immune cells in pancreatic cancer development and progression has been discussed elsewhere (Clark et al., 2009; Evans and Costello, 2012; Wachsmann et al., 2012; Vonderheide and Bayne, 2013). We refer readers interested in exhaustive summaries of the field to those reviews. We focus here on studies in human samples in which a correlation between immune infiltrates and clinicopathologic features of PDAC that have prognostic significance and an impact on patients' survival has been documented. These studies are summarized in Table 1.

**Table 1:** **Tumor infiltrating immune cells as predictors of the clinical outcome after surgery in pancreatic cancer patients**.

**Immune cells in the tumor**	**Predictive marker of favorable clinical outcome**	**References**
T cells	High CD4/8(^+/+^) counts^a^	Fukunaga et al., 2004
	CD4^+high^/CD8^+high^ counts^b^	Ino et al., 2013
	GATA-3^+^/T-bet^+^ TILs ratio below the median value^c^	De Monte et al., 2011
	CD4^+high^/CD8^+high^/%Treg^low^ counts^d^	Ino et al., 2013
Mast cells	Low counts^e^	Strouch et al., 2010
	Low counts in the intratumor border zone^f^	Cai et al., 2011
	Counts below the MCs score^g^	Chang et al., 2011
Macrophages	Low CD163^+^/CD204^+^ cells infiltration^h^	Kurahara et al., 2011
	Low FRβ^+^ macrophages infiltration^i^	Kurahara et al., 2012
	Low M2 macrophages (CD163^+^/CD204^+^ cells) infiltration^j^	Ino et al., 2013

a T cell counts were considered high for CD4^+^ ≥ 20 and CD8^+^ ≥ 100, corresponding to average numbers of 5 fields.

b High and low are based on the median values of CD4^+^ and CD8^+^ T cell counts.

c Patients were categorized in two groups based on the median value of the ratio of the percentage of GATA-3^+^/T-bet^+^ TILs.

d Patients were categorized based on the average values of CD4^+^ T cells and CD8^+^ T cell counts and of the percentage of Tregs.

e MCs counts were defined low if <8 and high if >13.

f Patients were categorized in two groups based on the median values of MCs counts.

g MCs score was set at 3.68 and it was defined as the ratio of the number of MCs to the percentage of CD45^+^ cells.

h Four grade infiltrations were considered: weak (<20/mm^2^), moderate (>20<40/mm^2^), strong (>40<60/mm^2^), and massive (>60/mm^2^). Low correspond to weak plus moderate; high correspond to strong plus massive.

i Patients were categorized in two groups based on the median values of FRβ^+^ macrophages counts.

j Patients were categorized in two groups based on the median values M2 macrophages counts.

## Tumor infiltrating lymphocytes

Tumor infiltrating lymphocytes (TILs) are present in several solid tumors and key features such as their distribution, density, and function dictate their anti-*versus* pro-tumor activity (Galon et al., 2006; Fridman et al., 2011, 2012). The major anti-tumor effectors are memory (CD45RO^+^) cytotoxic CD8^+^ T cells while more complex is the role of CD4^+^ T cells that depends on the pattern of cytokines produced. Several CD4^+^ T cell subsets have been described (Ruffell et al., 2010; Zhu et al., 2010): Th1 producing IFN-γ, Th2 producing IL-4, IL-5 and IL-13, Th17 producing IL-17, Th22 producing IL-22 and immunosuppressive Tregs. The role of the different subsets in tumor immunity is still under debate (Kennedy and Celis, 2008; Ruffell et al., 2010; Fridman et al., 2012).

In PDAC few studies have addressed the role of TILs in anti-tumor or pro-tumor activity and the correlation between those infiltrates and the clinical outcome.

The presence of TILs in PDAC was first reported in a study (Ademmer et al., 1998), which found that lymphocytes were typically localized as aggregates in the fibrotic interstitial tissue while very few cells reached the epithelial tumor cells. The amount of CD4^+^ and CD8^+^ T cells was variable among samples and showed a predominant CD45RO^+^ memory phenotype. Only few T cells were found in normal pancreatic tissue. Kalthoff and collaborators (Von Bernstorff et al., 2001) confirmed that TILs do not reach tumor cells in significant numbers, being “trapped” in the peritumoral tissue. Heterogeneous TILs distribution with both focal areas of high accumulation, mainly at the periphery of the tumor, and areas with diffusely scattered cells was reported in (Ryschich et al., 2005). In this study a survival analysis performed on 24 patients showed that median survival of patients with high density of CD8^+^ T cells was, although not statistically significant, considerably higher than that of the group with low CD8^+^ T cells density. Statistical significance was reached in Fukunaga et al. (2004), in which 80 patients were analyzed. The overall survival rate was significantly longer in patients with CD8^+^ but not CD4^+^ T cell infiltration and highest for patients positive for both T cells populations CD8/CD4(^+^/^+^) (positivity was defined as average counts from 5 fields ≥100 for CD8 and ≥20 for CD4). Interestingly, the Authors found a negative correlation between CD8/CD4(^+^/^+^) infiltration and both tumor depth and TNM stage and in multivariate analysis the CD8/CD4(^+^/^+^) infiltration was confirmed as an independent prognostic factor of survival.

The presence of FoxP3^+^CD4^+^CD25^+^ T regulatory cells (Tregs) possibly with immunosuppressive activity was evaluated in tumor tissues, inflammatory tissue and draining lymph nodes (LNs) in 198 PDAC patients and 15 patients with non-neoplastic lesions (Hiraoka et al., 2006). Tregs infiltration was localized in cancer stroma in areas of invasion. The prevalence of Tregs was significantly higher in PDAC than in inflammatory areas and in non-neoplastic lesions while no differences were observed in the LNs. When patients were divided into two groups based on values higher and lower than the average, the low Tregs group showed significantly better survival than the high Tregs group did. The Authors analyzed Tregs also in precursors lesions (PanIN) and interestingly found a significant increase of Tregs prevalence during progression from low-grade PanIN to invasive carcinoma (Hiraoka et al., 2006). In the same study intraepithelial CD8^+^ T cells inversely correlated with Tregs infiltration in the stroma: indeed they were present in PanIN-1, significantly decreased in PanIN-2 and drastically diminished to few in PanIN-3. More recently, in a large retrospective study of 212 tumor samples, the same Authors (Ino et al., 2013) reported that in multivariate analysis the prevalence of tumor infiltrating CD4^+^ T^high^/CD8^+^ T^high^/%Treg^low^ significantly correlated with longer survival and had a higher hazard ratio.

The characterization of TILs polarization in PDAC was initially reported in (Tassi et al., 2008), in which the presence of Th2, Th1 and Tregs cells in tumor samples was evaluated by immunohistochemistry using specific antibodies for GATA-3 (i.e., to detect Th2 cells), T-bet (i.e., to detect Th1 cells) and FoxP3. In this study PDAC patients undergoing surgery had circulating carcinoembryonic antigen-specific CD4^+^ Th2 cells in the presence of conserved anti-viral Th1 immunity. Furthermore, analysis of TILs in tumor samples from the same patients showed that, in agreement with the data in the blood, the number of lymphoid cells expressing GATA-3 was significantly superior to that of lymphoid cells expressing T-bet. FoxP3 was also expressed in lymphoid cells, as previously reported (Hiraoka et al., 2006), and cells expressing FoxP3 were in greater proportion relative to T-bet but in lower proportion relative to GATA-3. More recently, we analyzed 69 tumor samples and found that the amount of TILs differed among the samples and that in all but one case the percentage of GATA-3^+^ was significantly higher than that of T-bet^+^ TILs (De Monte et al., 2011). To compare samples with different amounts of TILs we used the ratio of the percentage of GATA-3^+^/T-bet^+^ TILs and performed survival analysis. We found that patients with a ratio inferior to the median value had a statistically prolonged survival. Multivariate analysis stratifying for tumor stage, grading, size, site, patient performance status, gender, age, surgical resection margins, postoperative CA19.9 value, and postoperative treatment confirmed that the ratio was independently predictive of both disease-free and overall survival. We also identified a complex cross-talk among tumor cells, CAFs and DCs that implicates (i) the secretion of pro-inflammatory cytokines (i.e., TNF-α and IL-1β) by tumor cells with (ii) activation of CAFs to secrete the thymic stromal lymphopoietin (TSLP), (iii) activation by CAFs-derived TSLP of resident DCs with Th2 polarizing capability and which secrete Th2 attracting chemokines, and (iv) migration of TSLP activated DCs, possibly tumor antigen-loaded, to draining LNs where Th2 cell priming occurs. Interestingly, epithelial cells derived TSLP was also correlated with the presence of Th2 inflammation in breast carcinoma (Pedroza-Gonzalez et al., 2011).

Collectively, intraepithelial CD8^+^ T cells infiltration is very rare in PDAC. CD4^+^ and CD8^+^ T cells are predominantly present in the stroma either dispersed or in aggregates, mainly at the periphery of the tumor. The prevalence of CD4^+^ T^high^/CD8^+^ T^high^/%Treg^low^ and the ratio of the percentage of GATA-3^+^/T-bet^+^ TILs in the tumor stroma were found to be independent predictive factors of overall survival after surgery in PDAC patients. An open issue remains as to the antigen-specificity of tumor infiltrating T cells.

## Mast cells

MCs have been extensively studied for their role in allergic and anaphylactic reactions during which FcεRI aggregation leads to degranulation and release of multiple mediators (Galli et al., 2005, 2008). They are also known to be critical players in inflammatory diseases where they act through “selective” release of mediators without degranulation (Theoharides et al., 2007).

MCs infiltration is a relevant component of the tumor microenvironment in a number of human malignancies (Theoharides et al., 2007). MCs accumulate in the tumor stroma in response to tumor-derived chemoattractants such as MCP-1 and RANTES. However, there is no general agreement on their role in cancer. Indeed, MCs counts were shown to correlate with either favorable or poor prognosis depending on the tumor (Theoharides and Conti, 2004; Khazaie et al., 2011; Ribatti and Crivellato, 2012). MCs can exert pro-tumorigenic effects by secreting factors like VEGF and IL-8 that promote tumor angiogenesis, tumor growth factors (i.e., PDGF, NGF, SCF) and proteases that facilitate metastases. On the other hand, high MCs counts in draining LNs were found to correlate with better prognosis in human breast cancer where a mechanism involving allergy-like degranulation with inhibitory effects on tumor cell growth was hypothesized (Theoharides and Conti, 2004). This dual role is tentatively explained with their different mechanisms of secretion: inflammation-driven selective secretion is pro-tumorigenic while allergy-like degranulation is anti-tumor.

In PDAC MCs were found in significant higher numbers in tumor tissue compared to normal pancreas (Esposito et al., 2002). MCs were located around ducts, blood vessels and nerves in the connective tissue without particular clustering around neoplastic cells. In a more extensive analysis of 137 patients, the same Authors (Esposito et al., 2004) also showed that MCs number correlated with the presence of LNs metastases and intratumor microvessel density. Moreover, patients with low numbers of infiltrating MCs compared with those with high numbers had a tendency toward a longer survival.

More recently, a study (Strouch et al., 2010) on 53 tumor specimens found that increased MCs infiltration correlated with higher-grade tumors. Recurrence-free and disease-specific survival was found significantly worse in patients with high MCs counts compared to those with low counts. Interestingly, patients with PDAC had higher serum tryptase activity than patients with benign disease. Furthermore, *in vitro* experiments using cell lines demonstrated a cross-talk between tumor cells that secrete MCs attracting factor(s) and MCs, which in turn release cancer cell growth and pro-invasive factor(s).

Particular attention to MCs distribution in different tumor areas was the focus of a study (Cai et al., 2011), which evaluated in 103 patients MCs infiltration in intratumoral and peritumoral areas and further in their border and center zones. In this correlative study the Authors found that in the intratumoral border zone, but not in the peritumoral or in the intratumoral center zone, high MCs counts were associated with LNs metastasis, tumor stage, lymphatic, and microvascular invasion. Significantly, high intratumoral border zone infiltration was identified as an independent prognostic factor of overall survival in resected patients, underlying the relevance of zone-specific distribution of MCs in PDAC.

High MCs infiltration was further confirmed as a negative predictive marker of survival in another study (Chang et al., 2011), which comprised 67 tumor samples. However, in multivariate analysis the MCs score used to stratify the patients did not reach statistical significance.

Collectively, the number of infiltrating MCs was found increased in PDAC compared to normal pancreas. A correlation between increased MCs numbers and the presence of LNs metastasis, tumor grade, intratumor microvessel density, and lymphatic and microvascular invasion was observed. The degree of MCs infiltration was identified as a predictive marker of patients' survival in the majority of studies.

## Macrophages

Solid tumors are frequently infiltrated by tumor-associated macrophages (TAMs), which are driven by tumor and T cell derived cytokines (especially IL-4, IL-10, and IL-13) to acquire an “alternatively activated” M2 phenotype with pro-tumor properties (Mantovani et al., 2002; Gordon, 2003). This M2-type macrophages are opposed to the “classic” M1-type that are activated by Th1 cytokines (IFN-γ, IL-1β) and are endowed with anti-tumor properties (Lewis and Pollard, 2006). TAMs receive signals from diverse cells within the tumor microenvironment and promote tumor growth and progression through regulation of angiogenesis, production of soluble mediators, which support proliferation, survival and invasive properties of tumor cells, and direct and indirect immunosuppression/modulation of lymphoid cells function (Mantovani et al., 2002; Qian and Pollard, 2010; Balkwill and Mantovani, 2012; Ruffell et al., 2012).

CD68^+^ cells were found increased in PDAC compared to normal pancreatic tissue: however, in 137 tumor samples no significant correlation with cumulative survival was demonstrated (Esposito et al., 2004). A more accurate analysis of TAM polarization was performed on 76 patients samples by immunohistochemistry using both anti-CD68 (i.e., pan macrophage) and anti-CD163 and anti-CD204 antibodies (i.e., which should preferentially stain M2-type macrophages) (Kurahara et al., 2011). The number of CD68^+^ cells varied among the samples examined: some tumors were extensively infiltrated while others had only sparse CD68^+^ cells infiltration. CD163^+^ and CD204^+^ cells were present within the same areas and the counts were lower than the number of total CD68^+^ cells. Interestingly, the number of CD163^+^ and CD204^+^ better then CD68^+^ cells correlated with LNs metastasis (Kurahara et al., 2011). Patients were stratified into two groups based on mean values of CD68^+^ or CD163^+^/CD204^+^ cell counts. The Authors found that lymphatic vessel density in the invasive front was significantly higher for high CD163^+^/CD204^+^ tumor samples compared to low samples but not statistically significant difference was found between the high and low CD68^+^ infiltration. The data suggests that increased M2-type infiltration in the invasive front might have a role in lymph-angiogenesis and lymphatic metastatic spread in PDAC. When the prognostic impact of TAMs infiltration was assessed, the prognosis was significantly poorer in the high CD163^+^/CD204^+^ group compared with the low. Whereas, although the high CD68^+^ tended to have a poor prognosis compared with the low CD68^+^, no significant difference in the survival rate between the high and low CD68^+^ cell counts was found.

In a following study (Kurahara et al., 2013), the same Authors showed a strong association among the density of VEGF-C expressing M2-type TAMs in regional LNs, nodal lymphatic vessel density and the incidence of isolated tumor cells in pN0 pancreatic cancer, further suggesting that M2-polarized TAMs may indeed facilitate nodal lymph-angiogenesis and promote lymph nodes micro-metastases.

Infiltration of macrophages that express the folate receptor β (FRβ) [i.e., a marker expressed in M2-type macrophages (Puig-Kroger et al., 2009)] was also investigated in PDAC (Kurahara et al., 2012). FRβ^+^ macrophages were prominent in perivascular areas of the tumor invasive front and when in high numbers they showed (i) a positive association with high tumor micro-vessel density, (ii) a high incidence of hematogenous metastasis, and (iii) poor prognosis in PDAC patients (Kurahara et al., 2012).

In agreement with an inverse correlation between M2-type TAMs infiltration and disease survival, a recent study (Ino et al., 2013), which included 212 patients, reported that high CD163^+^ and CD204^+^ cells infiltration was significantly associated with both shorter disease-free and overall survival. In the same study the presence of M2-type macrophages and the percentage of Tregs correlated with the presence of venous invasion.

Another study (Tjomsland et al., 2011) evaluated macrophage infiltration in PDAC samples by CD68 and CD163 gene expression analysis and found that, in contrast with the studies reported above, high CD163 expression correlated with longer survival. However, clinical correlation was done in a limited number of samples (30 patients) compared to the ones reported above (Kurahara et al., 2011, 2012, 2013; Ino et al., 2013) and based on CD163 gene expression rather then actual macrophage counts.

Collectively, higher numbers of CD68^+^ cells were found in PDAC samples compared to normal pancreas. Functional polarization toward M2-type correlates with a poor prognosis after surgery in resected patients. High CD163^+^ and CD204^+^ cell counts in perivascular areas of the tumor invasive front correlate with lymphangiogensis, high tumor micro-vessel density, LNs occult metastasis and poor prognosis in PDAC patients.

## Concluding remarks

Several studies have demonstrated that tumor antigens specific T cells are present in the circulation of PDAC patients (Laheru and Jaffee, 2005). However, the presence of a Th2-type inflammatory and immunosuppressive microenvironment questions the possibility that anti-tumor Th1 effectors reach the tumor and eventually maintain their effector functions. Future therapeutic approaches in PDAC should implement the efficacy of Th1 effectors by a combination of active and adoptive immunotherapy (Mellman et al., 2011) and strategies, such as the use of immunomodulators and/or therapy with agonistic CD40 that has proved to be efficacious in PDAC patients (Beatty et al., 2011), aimed at redirecting Th2 toward Th1-type inflammation in the tumor microenvironment. Moreover, since extensive immunohistochemical evaluations in bioptic material are not feasible, it will be interesting to compare the results of the studies reported here in surgical specimens from patients undergoing neoadjuvant therapies such as chemo or immunotherapy.

### Conflict of interest statement

The authors declare that the research was conducted in the absence of any commercial or financial relationships that could be construed as a potential conflict of interest.

## Abbreviations

CAFs: cancer associated fibroblasts
DCs: dendritic cells
FRβ: folate receptor β
LNs: lymph nodes
MCs: mast cells
PanIN: precursor lesions
PDAC: pancreatic ductal adenocarcinoma
TAMs: tumor associated macrophages
TILs: tumor infiltrating lymphocytes
Tregs: T regulatory cells
TSLP: thymic stromal lymphopoietin.
